# Evaluation of Ruthenium(II) *N*-Heterocyclic Carbene Complexes as Antibacterial Agents and Inhibitors of Bacterial Thioredoxin Reductase

**DOI:** 10.3390/molecules26144282

**Published:** 2021-07-15

**Authors:** Hilke Burmeister, Pascal Dietze, Lutz Preu, Julia E. Bandow, Ingo Ott

**Affiliations:** 1 Institute of Medicinal and Pharmaceutical Chemistry, Technische Universität Braunschweig, Beethovenstr. 55, 38106 Braunschweig, Germany; hilkeburmeister@web.de (H.B.); l.preu@tu-bs.de (L.P.); 2 Applied Microbiology, Faculty of Biology and Biotechnology, Ruhr University Bochum, Universitätsstr. 150, 44801 Bochum, Germany; Pascal.Dietze@ruhr-uni-bochum.de (P.D.); julia.bandow@rub.de (J.E.B.)

**Keywords:** antibacterial, *N*-heterocyclic carbenes, ruthenium, thioredoxin reductase

## Abstract

A series of ruthenium(II) complexes with *N*-heterocyclic carbene (NHC) ligands of the general type (arene)(NHC)Ru(II)X_2_ (where X = halide) was prepared, characterized, and evaluated as antibacterial agents in comparison to the respective metal free benzimidazolium cations. The ruthenium(II) NHC complexes generally triggered stronger bacterial growth inhibition than the metal free benzimidazolium cations. The effects were much stronger against Gram-positive bacteria (*Bacillus subtilis* and *Staphylococcus aureus*) than against Gram-negative bacteria (*Escherichia coli*, *Acinetobacter* *baumannii*, *Pseudomonas aeruginosa*), and all complexes were inactive against the fungus *Candida albicans*. Moderate inhibition of bacterial thioredoxin reductase was confirmed for selected complexes, indicating that inhibition of this enzyme might be a contributing factor to the antibacterial effects.

## 1. Introduction

Metal complexes with *N*-heterocyclic carbene (NHC) ligands represent an important type of catalysts, however, in addition to their application in chemistry, the biomedical properties have been evaluated intensively. In particular, inorganic medicinal chemistry and bioorganometallic chemistry nowadays make intensive use of the relatively high stability of the metal-carbon bonds of these compounds and the convenient synthetic access to a broad variety of NHC ligands [1,2,3,4,5,6,7].

The design of drug candidates based on metal NHC complexes has been dominated by gold and silver as metals, however, more recently, an increasing number of metals has demonstrated very promising results, including, for example, rhodium [8,9,10,11,12] and iridium [12,13] species.

We and others have recently reported on ruthenium(II) NHC complexes of the general type (*p*-cymene)(NHC)Ru(II)X_2_ (where X = halide) and structurally related complexes as novel anticancer drug candidates [14,15,16,17,18,19,20]. For complexes with the general structure (*p*-cymene)(NHC)Ru(II)Cl_2_ with different substituents on the NHC nitrogen atoms, the antiproliferative activity was dependent on the lipophilicity of the nitrogen side chains and on cellular uptake. The compounds inhibited thiol or selenol containing enzymes (e.g., thioredoxin reductase, cathepsin B), were generally reactive towards biologically relevant thiols, and underwent ligand exchange reactions (e.g., the replacement of the NHC ligand upon reaction with *N*-acetylcysteine could be confirmed by NMR). Interestingly, the complexes were well tolerated regarding toxicity in zebrafish embryos and mouse models [14,17]. Hartinger et al. demonstrated by means of X-ray crystallography with the model protein hen egg white lysozyme that, upon binding, the *p*-cymene ligand of the complexes was replaced, while the NHC ligand remained coordinated in this case [21]. Interestingly, the same group also demonstrated that derivatives with a pyridine in one of the side chain showed enhanced stability towards cysteine [19].

Notably, the probably first report on biological activity of (arene)(NHC)Ru(II)X_2_ complexes describes their application as antibacterial agents [22]. However, the literature on such application of the complexes appears limited to very few reports [22,23,24,25].

In order to shed more light on the potential of ruthenium NHC complexes as antibacterial agents, we synthesized and evaluated their bacterial cell growth inhibition. Furthermore, the inhibition of bacterial thioredoxin reductase was studied as a possible mechanism of action. The target compounds are based on the (*p*-cymene)(NHC)Ru(II)Cl_2_ scaffold of our previous work [14] and contain substituents with different inductive and mesomeric effects on the NHC moiety. The substituents are thus expected to influence the donor capacity of the NHC ligand, and with this, the reactivity and biological activity of the organometallics.

## 2. Synthesis and Characterization

**Scheme 1:**
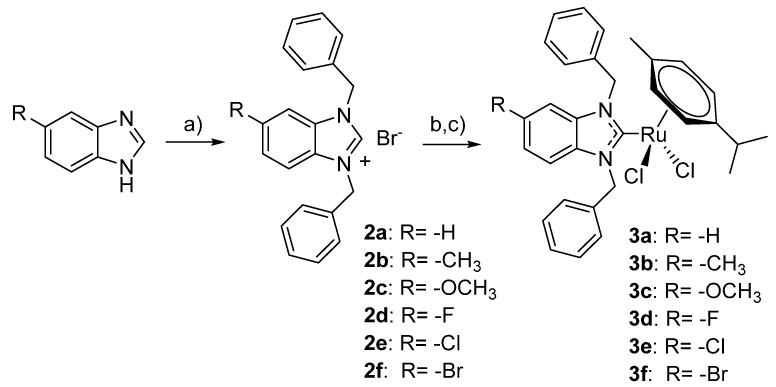
Synthesis of the (NHC)(*p*-Cym)Ru(II)Cl_2_ complexes; (a) K_2_CO_3_, benzyl bromide, (b) Ag_2_O, (c) [(*p*-Cym)RuCl_2_]_2._.

The (*p*-cymene)(NHC)Ru(II)Cl_2_ target compounds were obtained based on an established two-step procedure (Scheme 1). In the first step, the benzimidazoles **1a**–**1f** were alkylated with benzyl bromide resulting in the benzimidazolium bromides **2a**–**2f**. In the second step, the target compounds **3a**–**3f** were obtained in a one-pot reaction by activating **2a**–**2f** with silver oxide followed by transmetalation with [(*p*-Cym)RuCl_2_]_2_. The respective complexes required adjustments regarding the reaction period of the transmetalation reaction in step 2 and the isolation procedure. Thus, to obtain complexes **3d**–**3f** with halide substituents, extended reaction periods of up to 7 days were required. Complex **3f** was isolated by column chromatography over silica in contrast to the other complexes, which could be obtained in high purity by filtration over celite followed by crystallization.

The obtained target compounds were characterized by ^1^H- and ^13^C-NMR and mass spectroscopy. The high purities were confirmed by elemental analyses.

**Figure 1:**
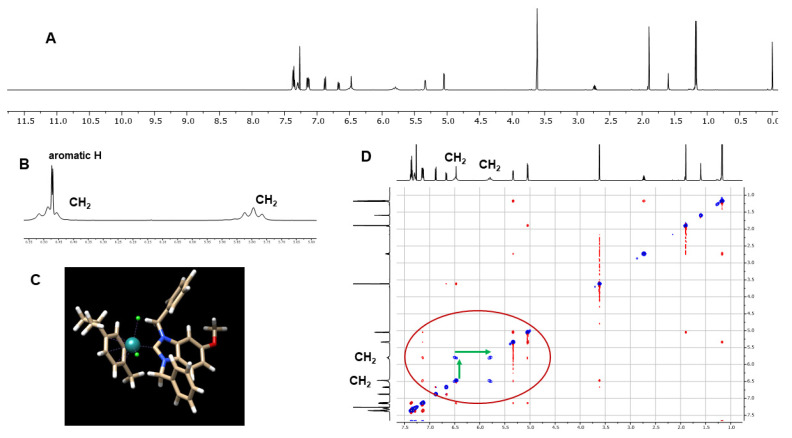
(**A**) ^1^H-NMR spectrum (600 MHz, CDCl_3_) of **3c**; (**B**) magnification of the section between 5.6 and 6.5 ppm; (**C**) DFT calculated geometry of **3c;** (**D**) NOESY spectrum (600 MHz, CDCl_3_) of **3c**, the EXSY correlation peaks between the CH_2_ hydrogens are marked with green arrows.

Several diagnostic features confirming complex formation can be observed in the NMR spectra. The hydrogen signal of the benzimidazole C2 carbon in the ^1^H-NMR spectra, which appears in the range of 11.6–12.1 ppm in the spectra of the benzimidazolium bromides **2a**–**2f**, is missing in the spectra of the metal complexes **3a**–**3f**. In the ^13^C-NMR spectra, the signal of the C2 carbon is shifted downfield from 142–144 ppm in the spectra of **2a**–**2f** to 192–194 ppm in the spectra of **3a**–**3f**. The CH_2_ groups of the benzyl side chains in the benzimidazolium cations **2a**–**2f** form the expected singlets in the ^1^H-NMR spectra. The respective CH_2_ groups in the complexes **3a**–**3f**, however, are non-equivalent, with diastereotopic protons and each individual hydrogen appears as a separate broadened signal (Figure 1A,B).

Figure 1 shows exemplarily the ^1^H-NMR spectrum of **3c** (Figure 1A) with the broadened CH_2_ signals (Figure 1B), which appear as pseudo-triplets due to the overlapping of two doublets. The calculated geometries of **3c** (Figure 1C) show that, for each CH_2_ group, only one individual hydrogen is directed towards a chlorido ligand, causing a different chemical environment for the individual protons of each CH_2_ group that results in a signal shift of approximately 0.7 ppm. Further confirmation was obtained by a NOESY/EXSY spectrum, which shows EXSY correlation signals as a consequence of the slow dynamic exchange process between the two individual hydrogens of each CH_2_ group (Figure 1D).

## 3. Antibacterial Activity and Inhibition of Bacterial TrxR

The antibacterial activity of compounds **2a**/**3a**–**2f**/**3f** was assessed in three Gram-negative (*Escherichia coli*, *Acinetobacter baumannii*, *Pseudomonas aeruginosa*) and in three Gram-positive strains (*Bacillus subtilis*, two different strains of *Staphylococcus aureus*) as minimal inhibitory concentration (MIC). The fungus *Candida albicans* was used as a non-bacterial reference organism. The broad-spectrum antibiotic Ciprofloxacin was used as a reference drug. The results are presented in Table 1.

The benzimidazolium cations **2a**–**2f** were inactive (MIC values > 1000 µM) against the Gram-negative bacteria as well as against *C. albicans*; however, low to moderate activity was recorded against the Gram-positive bacteria. Among them, compound **2c** was the most active with a MIC value of 19.5 µM against both *S. aureus* strains.

The ruthenium NHC complexes **3a**–**3f** caused very low activity against the Gram-negative bacteria (all MIC values above 200 µM) and were inactive against *C. albicans* (MIC values > 700 µM). Good activity was noted against all Gram-positive bacteria, with MIC values in the range of 11 to 53 µM. The most active complex was **3f**, for which an MIC value of 11.7 µM was recorded against *B. subtilis* and the methicillin and oxacillin resistant *S. aureus* ATCC 43300 strain. Comparing the metal free compounds **2a**–**2f** with the respective ruthenium(II) NHC complexes **3a**–**3f** confirms the activity-enhancing effect of the metal coordination. With the exception of the couple **2c**/**3c** all ruthenium complexes were substantially more active than the respective benzimidazolium derivatives.

The inhibition of TrxR has been considered as a novel mode of action for antibiotics and in our previous report, the relevance for gold NHC complexes had been shown [26]. In order to evaluate TrxR inhibition of ruthenium NHC complexes, complexes **3a**, **3b,** and **3f** were selected and the inhibition of purified *E. coli* TrxR was determined by the 5,5-dithiobis-(2-nitrobenzoic acid) reduction assay. The respective metal free benzimidazolium derivatives **2a**, **2b,** and **2f** were used as references and as expected were inactive (IC_50_ values > 100 µM). For the ruthenium NHC complexes **3a** and **3b** IC_50_ values of 24.8 (±5.4) µM (**3a**) and 14.0 (±1.6) µM (**3b**) were recorded, while **3f** was inactive (IC_50_ value > 100 µM). On the one hand, this confirms a moderate activity as bacterial TrxR inhibitors for ruthenium NHC complexes, but on the other hand, the missing efficacy as TrxR inhibitor of the strongest antibacterial complex **3f** shows that there is no direct correlation between bacterial TrxR inhibition and antibacterial activity.

## 4. Conclusions

A series of benzimidazolium cations and the corresponding organometallics of the type (*p*-cymene)(NHC)Ru(II)Cl_2_ were synthesized, characterized, and evaluated as antibacterial agents. The ruthenium(II) complexes were overall more active inhibitors of bacterial growth than the respective metal free benzimidazolium derivatives, confirming an activity enhancing effect of the metal coordination. Complex **3f** was the most active compound and exhibited a considerable selectivity for Gram-positive bacteria in comparison to Gram-negative bacteria or *C. albicans*. Such preference for Gram-positive bacteria is common due to the different characteristics of the bacterial cell wall of Gram-positive versus Gram-negative bacteria. In addition to that, many Gram-positive bacteria lack sufficient levels of glutathione and are highly dependent on an operating Trx/TrxR-system, making them very sensitive to strong TrxR inhibitors, such as gold compounds [26,27,28]. Accordingly, the ruthenium(II) NHC complexes were studied as inhibitors of bacterial TrxR. Moderate inhibition was noted for some complexes, however, there was no correlation to the antibacterial effects. Inhibition of TrxR is therefore unlikely a major mechanism of the antibacterial activity of the ruthenium NHC complexes, but might contribute to their biological profile in the sense of the complexes “hitting” multiple targets or disease pathways.

Further optimization of the efficacy of ruthenium(II) NHC complexes as antibacterial agents and studies on their mechanism of action are the subject of ongoing projects.

## 5. Materials and Methods

### 5.1. General

The reagents were purchased from Merck (Darmstadt, Germany), Thermo Fisher (Kandel, Germany), or TCI (Eschborn, Germany), and used without additional purification steps. All reactions were performed without precautions to exclude air or moisture. ^1^H and ^13^C NMR were recorded on a Bruker Biospin (Germany) DRX-400 AS (Germany), AVIIIHD 500, or AVII 600. A Finnigan MAT 95 was used to record the ESI mass spectra. The elemental analyses were performed using a Flash EA 1112 (Thermo Quest CE Instruments, Egelsbach, Germany). Absorption measurements for TrxR inhibition, and antiproliferative activity were performed on a Perkin-Elmer 2030 Multilabel Reader VICTOR^TM^ X4 (Rodgau, Germany). The synthesis of **2a** [29], **2c** [30], and **3a** [14] was reported. For computational chemistry, Gaussian 98 W (5.4 Rev A.9) was used with the B3LYP functional and LANL2DZ basis set [31].

### 5.2. Synthesis of the Benzimidazolium Bromides

The respective benzimidazole was dissolved in acetonitrile, treated with 2.5 equivalents K_2_CO_3_, and stirred at room temperature for 30 min. Two equivalents of benzyl bromide were added and stirring was continued until the reaction was complete as monitored by thin layer chromatography (periods are indicated below). The solvent was removed by reduced pressure; the residue was resuspended in dichloromethane and filtered. After evaporation under reduced pressure, the remaining oil was treated with diethyl ether to precipitate the product.

*1,3-Dibenzyl-5-methyl-1H-benzimidazolium bromide* (**2b**) prepared starting from 5-methyl-1*H*-benzimidazol (300.5 mg, 2.27 mmol), reaction period: 3 days; yield: 624.6 mg (1.59 mmol, 69.8%), white-yellowish powder; ^1^H-NMR (500 MHz, CDCl_3_-*d_1_*) δ = 11.83 (s, BeIm-H2, 1H), 7.52–7.48 (m, Bn^1^+Bn^2^-H2+H6, 4H), 7.44 (dd, ^3^*J*_H,H_ = 8.5 Hz, ^5^*J*_H,H_ = 0.7 Hz, BeIm-H7, 1H), 7.36 (m, Bn^1^+Bn^2^-H3-H5, 6H), 7.34 (m, BeIm-H4, 1H), 7.30 (dd, ^3^*J*_H,H_ = 8.6 Hz, ^4^*J*_H,H_ = 2.1 Hz, BeIm-H6, 1H), 5.85 (s, Bn^1^-CH_2_, 2H), 5.83 (s, Bn^2^-CH_2_, 2H), 2.45 (s, ^4^*J*_H,H_ = 0.7 Hz, BeIm-CH_3_, 3H); ^13^C-NMR (126 MHz, CDCl_3_-*d_1_*) δ = 142.55 (BeIm-C2), 138.09 (BeIm-C5_quart._), 132.60 (Bn^2^-C1_quart._), 132.56 (Bn^1^/Bn^2^-C1_quart._), 131.61 (BeIm-C7a_quart._), 129.39 (BeIm-C3a_quart._), 129.36 (Bn^1^/Bn^2^-C3-C5), 129.33 (Bn^1^/Bn^2^-C3-C5), 129.19 (Bn^1^/Bn^2^-C3-C5), 129.18 (Bn^1^/Bn^2^-C3-C5), 128.79 (BeIm-C6), 128.24 (Bn^1^/Bn^2^-C2+C6), 128.17 (Bn^1^/Bn^2^-C2+C6), 113.27 (BeIm-C7), 113.08 (BeIm-C4), 51.46 (Bn^1^-CH_2_), 51.24 (Bn^2^-CH_2_), 21.76 (BeIm-CH_3_); elemental analysis for C_22_H_21_BrN_2_ (theoreticalfound [%]): C (67.18/67.08), H (5.38/5.55), N (7.12/7.10); MS (ESI): *m*/*z* 313.17 [M − Br]^+^, 493.26 [M + 2H_2_O + 2MeOH]^+^, 707.26 [M + M – Br + 2H^+^]^+^.

*1,3-Dibenzyl-5-methoxy-1H-benzimidazolium bromide* (**2c**) [30] prepared starting from 5-methoxy-1*H*-benzimidazole (250.0 mg, 1.69 mmol); reaction period: 3 days; yield: 131.7 mg (0.32 mmol, 19.1%), white-grey powder; ^1^H-NMR (500 MHz, CDCl_3_-*d_1_*) δ = 11.62 (s, BeIm-H2, 1H), 7.53 (m, Bn^1^-H2+H6, 2H), 7.48 (m, Bn^2^-H2+H6, 2H), 7.43 (dd, ^3^*J*_H,H_ = 9.1 Hz, ^5^*J*_H,H_ = 0.6 Hz, BeIm-H7, 1H), 7.36 (m, Bn^2^-H3-H5, 3H), 7.33 (m, Bn^1^-H3-H5, 3H), 7.05 (dd, ^3^*J*_H,H_ = 9.1 Hz, ^4^*J*_H,H_ = 2.3 Hz, BeIm-H6, 1H), 7.02 (dd, ^4^*J*_H,H_ = 2.3 Hz, ^5^*J*_H,H_ = 0.6 Hz, BeIm-H4, 1H), 5.86 (s, Bn^2^-CH_2_, 2H), 5.81 (s, Bn^1^-CH_2_, 2H), 3.80 (s, BeIm-OCH_3_, 3H); ^13^C-NMR (126 MHz, CDCl_3_-*d_1_*) δ = 159.3 (BeIm-C5_quart._), 141.9 (BeIm-C2), 132.61 (Bn^2^-C1_quart._), 132.55 (Bn^1^-C1_quart._), 132.54 (BeIm-C7a_quart._), 129.31 (Bn^1^+Bn^2^-C2-C4), 129.29 (Bn^1^+Bn^2^-C2-C4), 129.18 (Bn^1^+Bn^2^-C2-C4), 129.14 (Bn^1^+Bn^2^-C2-C4), 128.30 (Bn^2^-C1,C5), 128.19 (Bn^1^-C1,C5), 125.34 (BeIm-C3a_quart._), 117.21 (BeIm-C6), 114.4 (BeIm-C7), 96.18 (BeIm-C4), 56.2 (BeIm-OCH_3_), 51.5 (Bn^1^-CH_2_), 51.2 (Bn^2^-CH_2_); elemental analysis for C_22_H_21_BrN_2_O (theoretical/found [%]): C (64.56/64.82), H (5.17/5.14), N (6.84/6.78); MS (ESI): *m*/*z* 329.2 [M − Br]^+^, 509.26 [M + 2H_2_O + 2MeOH]^+^, 737.25 [M + M − Br]^+^.

*1,3-Dibenzyl-5-fluoro-1H-benzimidazolium bromide* (**2d**) prepared starting from 5-fluoro-1*H*-benzimidazole (269.5, 1.98 mmol); reaction period: 10 days; yield: 432.0 mg (1.09 mmol, 54.9%), grey powder; ^1^H-NMR (500 MHz, CDCl_3_-*d_1_*) δ = 11.98 (s, BeIm-H2, 1H), 7.55 (dd, ^3^*J*_H,F_ = 4.4 Hz, ^4^*J*_H,H_ = 1.7 Hz, BeIm-H6, 1H), 7.54–7.50 (m, Bn^1^/Bn^2^-H2-H6, 4H), 7.41–7.33 (m, Bn^1^/Bn^2^-H2-H6, 6H), 7.24–7.21 (m, BeIm-H7, 1H), 7.20 (d, ^3^*J*_H,F_ = 2.0 Hz, BeIm-H4, 1H), 5.91 (s, Bn^1^/Bn^2^-CH_2_, 2H), 5.86 (s, Bn^1^/Bn^2^-CH_2_, 2H); ^13^C NMR (126 MHz, CDCl_3_-*d_1_*) δ = 161.14 (d, ^1^*J*_C,F_ = 249.7 Hz, BeIm-C5_quart._), 144.39 (BeIm-C2), 132.21 (Bn^1^-C1_quart._), 132.02 (Bn2-C1_quart._), 131.93 (BeIm-C7a_quart._), 129.52 (Bn^1^/Bn^2^-C2-C6), 129.46 (Bn^1^/Bn^2^-C2-C6), 129.40 (Bn^1^/Bn^2^-C2-C6), 128.34 (Bn^1^/Bn^2^-C2-C6), 128.33 (Bn^1^/Bn^2^-C2-C6), 127.82 (BeIm-C3a_quart._), 116.17 (d, ^3^*J*_C,F_ = 26.2 Hz, BeIm-C4), 115.39 (d, ^4^*J*_C,F_ = 10.1 Hz, BeIm-C7), 100.77 (d, ^3^*J*_C,F_ = 28.3 Hz, BeIm-C6), 51.93 (Bn^1^-CH_2_), 51.90 (Bn^2^-CH_2_); elemental analysis for C_21_H_18_BrFN_2_ (theroretical/found [%]): C (63.49/63.22), H (4.57/4.55), N (7.05/6.64); MS (ESI): *m*/*z* 317.14 [M − Br]^+^, 497.24 [M + 2H_2_O + 2MeOH]^+^, 713.21 [M + M − Br]^+^.

*1,3-Dibenzyl-5-chloro-1H-benzimidazolium bromide* (**2e**) prepared starting from 5-chloro-1*H*-benzimidazole (299.3 mg, 1.97 mmol); reaction period: 21 days; yield: 306.0 mg (0.74 mmol, 37.7%), white-yellowish powder; ^1^H NMR (500 MHz, CDCl_3_-*d_1_*) δ = 12.00 (s, BeIm-H2, 1H), 7.53 (dd, ^4^*J*_H,H_ = 2.0 Hz, ^3^*J*_H,H_ = 1.0 Hz, BeIm-H6, 1H), 7.51 (m, Bn^1^/Bn^2^-H2-H6, 4H), 7.50 (dd, ^3^*J*_H,H_ = 7.7, ^5^*J*_H,H_ = 0.6 Hz, BeIm-H7, 1H), 7.43 (dd, ^3^*J*_H,H_ = 8.9 Hz, ^4^*J*_H,H_ = 1.8 Hz, BeIm-H4, 1H), 7.41–7.33 (m, Bn^1^/Bn^2^-H2-H6, 6H), 5.90 (s, Bn^1^-CH_2_, 2H), 5.85 (s, Bn^2^-CH_2_, 2H); ^13^C NMR (126 MHz, CDCl_3_-*d_1_*) δ = 144.32 (BeIm-C2), 133.51 (BeIm-C7a_quart._), 132.17 (Bn^1^-C1_quart._), 132.03 (Bn^2^-C1_quart._), 132.02 (BeIm-C3a_quart._), 129.94 (BeIm-C5_quart._), 129.54 (Bn^1^/Bn^2^-C2-C6), 129.49 (Bn^1^/Bn^2^-C2-C6), 129.47 (Bn^1^/Bn^2^-C2-C6), 129.42 (Bn^1^/Bn^2^-C2-C6), 128.34 (Bn^1^/Bn^2^-C2-C6), 128.30 (Bn^1^/Bn^2^-C2-C6), 127.99 (BeIm-C4), 114.96 (BeIm-C6), 113.68 (BeIm-C7), 51.94 (Bn^1^-CH_2_), 51.83 (Bn^2^-CH_2_); elemental analysis for C_21_H_18_BrClN_2_ (theoretical/found [%]): C (60.96/60.62), H (4.39/4.56), N (6.77/6.49); MS (ESI): *m*/*z* 333.12 [M − Br]^+^, 513.21 [M + 2H_2_O + 2MeOH]^+^, 747.15 [M + M − Br]^+^.

*1,3-Dibenzyl-5-bromo-1H-benzimidazolium bromide* (**2f**) prepared starting from 5-Bromo-1*H*-benzimidazol (298.4 mg, 1.51 mmol); reaction period: 21 days; yield: 182.1 mg (0.40 mmol, 26.2%), orange powder; ^1^H-NMR (500 MHz, CDCl_3_-*d_1_*) δ = 11.98 (s, BeIm-H2, 1H), 7.68 (dd, ^4^*J*_H,H_ = 1.7 Hz, ^5^*J*_H,H_ = 0.6 Hz, BeIm-H4, 1H), 7.58 (dd, ^3^*J*_H,H_ = 8.9 Hz, ^4^*J*_H,H_ = 1.7 Hz, BeIm-H6, 1H), 7.52 (t, ^4^*J*_H,H_ = 1.8 Hz, Bn2-H3+H5, 2H), 7.51 (dd, ^4^*J*_H,H_ = 1.9 Hz, Bn^1^-H3+H5, 2H), 7.46 (dd, ^3^*J*_H,H_ = 8.9 Hz, ^5^*J*_H,H_ = 0.6 Hz, BeIm-H7, 1H), 7.42–7.38 (m, Bn^2^-H2+H4+H6, 3H), 7.38–7.34 (m, Bn^2^-H2+H4+H6, 3H), 5.89 (s, Bn^1^-CH_2_, 2H), 5.84 (s, Bn^2^-CH_2_ 2H); ^13^C-NMR (126 MHz, CDCl_3_-*d_1_*) δ = 144.06 (BeIm-C2), 132.38 (BeIm-C7a_quart._), 132.16 (Bn^1^-C1_quart._), 132.01 (Bn^2^-C1_quart._), 130.66 (BeIm-C6), 130.33 (BeIm-C3a_quart._), 129.56 (Bn^1^+Bn^2^-C2+C4+C6), 129.51 (Bn^1^+Bn^2^-C2+C4+C6), 129.47 (Bn^+^Bn^2^-C2+C4+C6), 129.43 (Bn^1^+Bn^2^-C2+C4+C6), 128.35 (Bn^1^+Bn^2^-C3+C5), 128.30 (Bn^1^+Bn^2^-C3+C5), 120.81 (BeIm-C5_quart._), 116.63 (BeIm-C4), 115.22 (BeIm-C7), 51.90 (Bn^1^-CH_2_), 51.78 (Bn^2^-CH_2_); elemental analysis for C_21_H_18_Br_2_N_2_ (theoretical/found [%]): C (55.05/55.31), H (3.96/3.72), N (6.11/5.95); MS (ESI): *m*/*z* 377.10 [M − Br]^+^.

### 5.3. Synthesis of Ru(II) NHC Complexes

The Ru(II) NHC complexes were prepared based on a reported procedure [14], which was optimized to achieve higher yields. The benzimidazolium bromides **2a**–**2f** were dissolved in dichloromethane, 0.6 equivalents of silver oxide were added, and the mixture was stirred for 12 h at room temperature. The Ru(II)-dimer [(*p*-Cym)RuCl_2_]_2_ (0.5 equivalents) was added and stirring was continued for 2–7 days at room temperature (see the reaction periods below). The progress of the reaction was monitored by thin-layer chromatography (eluent: 5% methanol in dichloromethane). Precipitated silver halogenide was removed by filtration over celite, the volume of the filtrate was reduced under vacuum to approximately 1 mL, and the same volume of ethyl acetate was added. Within 2 days red, crystals were formed. Further purification steps (if any) are mentioned below.

*(1,3-Dibenzyl-5-methyl-1H-benzimidazol-2-ylidene)-dichlorido-(η^6^-p-cymene)ruthenium(II)* (**3b**) prepared starting from 1,3-dibenzyl-5-methyl-1*H*-benzimidazoliumbromide (132.9 mg, 0.34 mmol), reaction period with [(*p*-Cym)RuCl_2_]_2_: 48 h yield: 138.4 mg (0.22 mmol, 66%), red crystals; ^1^H-NMR (600 MHz, CDCl_3_-*d_1_*) δ = 7.39–7.33 (m, Bn^1^+Bn^2^-H3+H5, 4H), 7.32–7.27 (m, Bn^1^+Bn^2^-H4, 2H), 7.14–7.10 (m, Bn^1^+Bn^2^-H2+H6, 4H), 6.89 (d, ^4^*J*_H,H_ = 1.0 Hz, BeIm-H4+H7, 2H), 6.83 (m, BeIm-H6, 1H), 6.60 (d, ^2^*J*_H,H_ = 17.2 Hz, Bn^1^-CH_2_, 1H), 6.51 (d, ^2^*J*_H,H_ = 17.1 Hz, Bn^2^-CH_2_, 1H), 5.81 (d, ^2^*J*_H,H_ = 17,1 Hz, Bn^2^-CH_2_, 1H), 5.73 (d, ^2^*J*_H,H_ = 17.2 Hz, Bn^1^-CH_2_, 1H), 5.33 (d, ^3^*J*_H,H_ = 5.4 Hz, *p*-Cym-H3+H5, 2H), 5.04 (d, ^3^*J*_H,H_ = 5.7 Hz, *p*-Cym-H2+H6, 2H), 2.70 (hept, ^3^*J*_H,H_ = 6.9 Hz, *p*-Cym-CH, 1H), 2.30 (d, ^4^*J*_H,H_ = 1 Hz, BeIm-CH3, 3H), 1.87 (s, *p*-Cym-CH3, 3H), 1.16 (d, ^3^*J*_H,H_ = 7.0 Hz, *p*-Cym-CH-CH3, 6H); ^13^C-NMR (151 MHz, CDCl_3_-*d_1_*) δ = 190.59 (BeIm-C2_quart._), 137.69 (Bn^1^-C1_quart._), 137.57 (Bn^2^-C1_quart._), 135.91(BeIm-C3a_quart._), 133.76 (BeIm-C7a_quart._), 133.30 (BeIm-C5_quart._), 128.92 (Bn^1^/Bn^2^-C3/C5), 128.87 (Bn^1^/Bn^2^-C3/C5), 127.41 (Bn^1^/Bn^2^-C2), 127.39 (Bn^1^/Bn^2^-C2), 125.95 (Bn^1^/Bn^2^-C2/C6), 125.80 (Bn^1^/Bn^2^-C2/C6), 124.46 (BeIm-C4), 111.44 (BeIm-C6), 111.22 (BeIm-C7), 107.73 (*p*-Cym-C4_quart._), 97.12 (*p*-Cym-C1_quart._), 85.31 (*p*-Cym-C3+C5), 84.61 (*p*-Cym-C2+C6), 52.96 (Bn^2^-CH_2_), 52.56 (Bn^1^-CH_2_), 30.58 (*p*-Cym-CH), 22.54 (*p*-Cym-CH-CH_3_), 22.42 (*p*-Cym-CH-CH_3_), 21.41 (BeIm-CH3), 18.21 (*p*-Cym-CH_3_); elemental analysis C_32_H_34_Cl_2_N_2_Ru (theoretical/found [%]): C (62.13/62.29), H (5.54/5.58) N (4.53/4.50); MS (ESI): *m*/*z* 583.15 [M − Cl]^+^, 548.19 [M − 2Cl]^+^, 413.06 [M − 2Cl – Cym − H]^+^.

*(1,3-Dibenzyl-5-methoxy-1H-benzimidazol-2-ylidene)-dichlorido-(η^6^-p-cymene)ruthenium(II)* (**3c**) prepared starting from 1,3-dibenzyl-5-methoxy-1*H*-benzimidazoliumbromide (65.0 mg, 0.16 mmol), reaction period with [(*p*-Cym)RuCl_2_]_2_: 48 h, yield: 49.7 mg (0.08 mmol, 49.3%), orange-red crystals; ^1^H-NMR (600 MHz, CDCl_3_-*d_1_*) δ = 7.36 (m, Bn^1^/Bn^2^-H2+H6, 4H), 7.32–7.27 (m, 2H, Bn^1^/Bn^2^-H4, 2H), 7.16–7.11 (m, Bn^1^/Bn^2^-H3+H5, 4H), 6.88 (d, ^3^*J*_H,H_ = 8.9 Hz, BeIm-H7, 1H), 6.67 (dd, ^3^*J*_H,H_ = 8.9 Hz, ^4^*J*_H,H_ = 2.3 Hz, BeIm-H6, 1H), 6.47 (d, ^4^*J*_H,H_ = 2.3 Hz, BeIm-H4, 1H), 6.57 (m, Bn^2^-CH_2_, 1H), 6.52 (m, Bn^1^-CH_2_, 1H), 5.83 (m, Bn^1^-CH_2_, 1H), 5.80 (m, Bn^2^-CH_2_, 1H), 5.34 (d, ^3^*J*_H,H_ = 6.0 Hz, *p*-Cym-H3+H5, 2H), 5.05 (d, ^3^*J*_H,H_ = 6.0 Hz, *p*-Cym-H2+H6, 2H), 3.62 (s, BeIm-OCH_3_, 3H), 2.73 (hept, ^3^*J*_H,H_ = 7.0 Hz, *p*-Cym-CH, 1H), 1.90 (s, *p*-Cym-CH_3_, 3H), 1.17 (d, ^3^*J*_H,H_ = 7.0 Hz, *p*-Cym-CH-CH_3_, 6H); ^13^C-NMR (151 MHz, CDCl_3_-*d_1_*) δ = 190.25 (BeIm-C2_quart._), 156.46 (BeIm-C5_quart._), 137.52 (Bn^1^-C1_quart._), 137.36 (Bn^2^-C1_quart._), 136.42 (BeIm-C7a_quart._), 130.18 (BeIm-C3a_quart._), 128.93 (Bn^1^/Bn^2^-C2+C6), 128.88 (Bn^1^/Bn^2^-C2+C6), 127.49 (Bn^1^/Bn^2^-C4), 127.46 (Bn^1^/Bn^2^-C4), 126.03 (Bn^1^/Bn^2^-C3+C5), 126.00 (Bn^1^/Bn^2^-C3+C5), 112.14 (BeIm-C6), 111.50 (BeIm-C7), 107.90 (*p*-Cym-C4_quart._), 97.22 (*p*-Cym-C1_quart._), 95.73 (BeIm-C4), 85.36 (*p*-Cym-C3+C5), 84.51 (*p*-Cym-C2+C6), 55.73 (BeIm-OCH_3_), 53.05 (Bn^1^-CH_2_), 52.86 (Bn^2^-CH_2_), 30.60 (*p*-Cym-CH), 22.48 (*p*-Cym-CH-CH_3_), 18.29 (*p*-Cym-CH_3_); elemental analysis C_32_H_34_Cl_2_N_2_ORu (theoretical/found [%]): C (60.57/60.95), H (5.40/5.40) N (4.41/4.41); MS (ESI): *m*/*z* 599.14 [M − Cl], 465.03 [M – Cl − Cym], 429.05 [M − 2Cl – Cym − H], 329.17 [M − 2Cl – Cym − Ru].

*(1,3-Dibenzyl-5-fluoro-1H-benzimidazol-2-ylidene)-dichlorido-(η^6^-p-cymene)ruthenium(II)* (**3d**) prepared starting from 1,3-dibenzyl-5-fluoro-1*H*-benzimidazolium bromide (130.5 mg, 0.33 mmol), reaction period with [(*p*-Cym)RuCl_2_]_2_: 4 days; yield: 100.2 mg (0.18 mmol, 49%), orange powder; ^1^H-NMR (500 MHz, CDCl_3_-*d_1_*) δ = 7.41–7.34 (m, Bn^1^/Bn^2^-H2+H6, 4H), 7.31 (m, Bn^1^/Bn^2^-H3-H5, 2H), 7.15–7.09 (m, Bn^1^/Bn^2^-H3-H5, 4H), 6.93 (dd, ^3^*J*_H,H_ = 8.9 Hz, ^4^*J*_H,F_ = 4.3 Hz, BeIm-H7, 1H), 6.81 (td, ^3^*J*_H,H_ = 8.9 Hz, ^3^*J*_H,F_ = 5.8 Hz, ^4^*J*_H,H_ = 2.3 Hz, BeIm-H6, 1H), 6.71 (dd, ^3^*J*_H,F_ = 8.3 Hz, ^4^*J*_H,H_ = 2.3 Hz, BeIm-H4, 1H), 6.53 (s, Bn^1^/Bn^2^-CH_2_, 1H), 6.50 (s, Bn^1^/Bn^2^-CH_2_, 1H), 5.84 (d, ^2^*J*_H,H_ = 18.4 Hz, Bn^1^/Bn^2^-CH_2_, 1H), 5.78 (d, ^2^*J*_H,H_ = 17.7 Hz, Bn^1^/Bn^2^-CH_2_, 1H), 5.36 (d, ^3^*J*_H,H_ = 5.9 Hz, *p*-Cym-H3+H5, 2H), 5.08 (d, ^3^*J*_H,H_ = 5.9 Hz, *p*-Cym-H2+H6, 2H), 2.73 (hept, ^3^*J*_H,H_ = 6.9 Hz, *p*-Cym-CH, 1H), 1.91 (s, *p*-Cym-CH_3_, 3H), 1.18 (d, ^3^*J*_H,H_ = 6.9 Hz, *p*-Cym-CH-CH_3_, 6H); ^13^C-NMR (126 MHz, CDCl_3_-*d_1_*) δ = 193.72 (BeIm-C2), 159.24 (d, *J* = 243.2 Hz, BeIm-C5), 137.11 (Bn^1^/Bn^2^-C1_quart._), 136.81 (Bn^1^/Bn^2^-C1_quart._), 135.92 (d, *J* = 12.2 Hz, BeIm-C7a_quart._), 132.03 (BeIm-C3a_quart._), 129.07 (Bn^1^/Bn^2^-C2+C6), 129.01 (Bn^1^/Bn^2^-C2+C6), 127.73 (Bn^1^/Bn^2^-C3-C5), 127.65 (Bn^1^/Bn^2^-C3-C5), 125.92 (Bn^1^/Bn^2^-C3-C5), 125.90 (Bn^1^/Bn^2^-C3-C5), 112.35 (d, *J* = 9.8 Hz, BeIm-C7), 111.21 (d, *J* = 25.5 Hz, BeIm-C6), 108.18 (*p*-Cym-C4_quart._), 98.84 (d, *J* = 27.7 Hz, BeIm-C4), 97.30 (*p*-Cym-C1_quart._), 85.45 (*p*-Cym-C3+C5), 84.79 (*p*-Cym-C2+C6), 53.27 (Bn^1^/Bn^2^-CH_2_), 53.18 (Bn^1^/Bn^2^-CH_2_), 30.64 (*p*-Cym-CH), 22.45 (*p*-Cym-CH-CH_3_), 18.31 (*p*-Cym-CH_3_); elemental analysis for C_31_H_31_Cl_2_FN_2_Ru (theoretical/found [%]): C (59.81/59.31), H (5.02/5.08) N (4.50/4.45); MS (ESI): *m*/*z* 587.12 [M − Cl], 552.16 [M − 2Cl], 417.03 [M − 2Cl − *p* − Cym − H].

*(1,3-Dibenzyl-5-chloro-1H-benzimidazol-2-ylidene)-dichlorido-(η^6^-p-cymene)ruthenium(II)* (**3e**) prepared starting from 1,3-dibenzyl-5-chloro-1*H*-benzimidazolium bromide (90.5 mg, 0.22 mmol), reaction period with [(*p*-Cym)RuCl_2_]_2_: 7 days, yield: 71.6 mg (0.11 mmol, 51%), orange-brown crystals; ^1^H-NMR (500 MHz, CDCl_3_-*d_1_*) δ = 7.41–7.36 (m, Bn^1^/Bn^2^,H3-H5, 3H), 7.35–7.28 (m, Bn^1^/Bn^2^,H3-H5, 3H), 7.12–7.08 (m, Bn^1^/Bn^2^,H2+H6, 4H), 7.05 (dd, ^3^*J*_H,H_ = 8.7 Hz, ^4^*J*_H,H_ = 1.9 Hz, BeIm-H6, 1H), 7.00 (dd, ^4^*J*_H,H_ = 1.9 Hz, ^5^*J*_H,H_ = 0.5 Hz, BeIm-H4, 1H), 6.92 (dd, ^3^*J*_H,H_ = 8.7 Hz, ^5^*J*_H,H_ = 0.5 Hz, BeIm-H7, 1H), 6.54 (m, Bn^1^-CH_2_, 1H), 6.51 (m, Bn^2^-CH_2_, 1H) 5.84 (d, ^2^*J*_H,H_ = 17.2 Hz, Bn^2^-CH_2_, 1H), 5.75 (d, ^2^*J*_H,H_ = 17.2 Hz, Bn^1^-CH_2_, 1H), 5.38–5.32 (m, *p*-Cym-H3+H5, 2H), 5.07 (d, *J* = 6.1 Hz, *p*-Cym-H3+H5, 2H), 2.71 (hept, *J* = 7.0 Hz, *p*-Cym-CH, 1H), 1.89 (s, *p*-Cym-CH_3_, 3H), 1.17 (d, *J* = 6.9 Hz, *p*-Cym-CH-CH_3_, 6H); ^13^C-NMR (126 MHz, CDCl_3_-*d_1_*) δ = 194.18 (BeIm-C2, 1C), 137.02 (Bn2-C1_quart._), 136.88 (Bn1-C1_quart._), 136.16 (BeIm-C7a_quart._), 134.14 (BeIm-C5), 129.35 (BeIm-C3a_quart._), 129.12 (Bn^1^/Bn^2^-C3-C5), 129.03 (Bn^1^/Bn^2^-C3-C5), 127.75 (Bn^1^/Bn^2^-C3-C5), 127.68 (Bn^1^/Bn^2^-C3-C5), 125.88 (Bn^1^/Bn^2^-C2+C6), 125.78 (Bn^1^/Bn^2^-C2+C6), 123.70 (BeIm-C6), 112.45 (BeIm-C7), 111.56 (BeIm-C4), 108.19 (*p*-Cym-C4_quart._), 97.36 (*p*-Cym-C1_quart._), 85.49 (*p*-Cym-C3+C5), 84.86 (*p*-Cym-C2+C6), 53.23 (Bn^2^-CH_2_), 52.99 (Bn^1^-CH_2_), 30.63 (*p*-Cym-CH), 22.45 (*p*-Cym-CH-CH_3_), 18.27 (*p*-Cym-CH_3_); elemental analysis for C_31_H_31_Cl_3_N_2_Ru (theoretical/found [%]): C (58.27/58.33), H (4.89/4.84) N (4.38/4.26); MS (ESI): *m*/*z* 603.09 [M − Cl], 1243.15 [M + M − Cl + 2H], 433.00 [M − 2Cl − *p* − Cym − H], 333.12 [M − 2Cl − *p* − Cym − Ru].

*(1,3-Dibenzyl-5-bromo-1H-benzimidazol-2-ylidene)-dichlorido-(η^6^-p-cymene)ruthenium(II)* (**3f**) prepared starting from 1,3-dibenzyl-5-bromo-1*H*-benzimidazolium bromide (84.0 mg, 0.18 mmol), reaction period with [(*p*-Cym)RuCl_2_]_2_: 7 days; additional purification: column chromatography over silica (eluent: 5% methanol in dichloromethane), evaporation, and recrystallization (dichloromethane/n-hexane); yield: 20.5 mg (0.03 mmol, 16%), orange-red powder; ^1^H-NMR (600 MHz, CDCl_3_-*d_1_*) δ = 7.41–7.38 (m, Bn^2^-H2+H6, 2H), 7.38–7.35 (m, Bn^1^-H2+H6, 2H), 7.34–7.28 (m, Bn^1^+Bn^2^-H4, 2H), 7.19 (dd, ^3^*J*_H,H_ = 8.6 Hz, ^4^*J*_H,H_ = 1.7 Hz, BeIm-H6, 1H), 7.15 (m, ^4^*J*_H,H_ = 1.7 Hz, ^5^*J*_H,H_ = 0.45 Hz, BeIm-H4, 1H), 7.12–7.10 (m, Bn^1^/Bn^2^-H3+H5, 2H), 7.09–7.08 (m, Bn^1^/Bn^2^-H3+H5, 2H), 6.87 (m, ^3^*J*_H,H_ = 8.8 Hz, ^5^*J*_H,H_ = 0.45 Hz, BeIm-H7, 1H), 6.58 (d, ^2^*J*_H,H_ = 17.2 Hz, Bn^2^-CH_2_, 1H), 6.52 (d, ^2^*J*_H,H_ = 17.1 Hz, Bn^1^-CH_2_, 1H), 5.85 (d, ^2^*J*_H,H_ = 17.1 Hz, Bn^1^-CH_2_, 1H), 5.73 (d, ^2^*J*_H,H_ = 17.2 Hz, Bn^2^-CH_2_, 1H), 5.35 (d, ^3^*J*_H,H_ = 8.9 Hz, *p*-Cym-H3+H5, 2H), 5.06 (d, ^3^*J*_H,H_ = 5.9 Hz, *p*-Cym-H2+H6, 2H), 2.75–2.66 (m, ^3^*J*_H,H_ = 7.2 Hz, *p*-Cym-CH, 1H), 1.88 (s, *p*-Cym-CH_3_, 3H), 1.17 (d, ^3^*J*_H,H_ = 7.2 Hz, *p*-Cym-CH-CH_3_, 6H); ^13^C-NMR (151 MHz, CDCl_3_-*d_1_*) δ = 194.09 (BeIm-C2), 137.01 (Bn^1^-C1_quart._), 136.88 (Bn^2^-C1_quart._), 136.48 (BeIm-C7a_quart._), 134.49 (BeIm-C3a_quart._), 129.12 (Bn^2^-C2+C6), 129.02 (Bn^1^-C2+C6), 127.74 (Bn^1^/Bn^2^-C4), 127.65 (Bn^1^/Bn^2^-C4), 126.40 (BeIm-C6), 125.85 (Bn^1^/Bn^2^-C3+C5), 125.74 (Bn^1^/Bn^2^-C3+C5), 116.69 (BeIm-C5), 114.36 (BeIm-C4), 112.86 (BeIm-C7), 108.16 (*p*-Cym-C4_quart._), 97.34 (*p*-Cym-C1_quart._), 85.47 (*p*-Cym-C3+C5), 84.86 (*p*-Cym-C2+C6), 53.18 (Bn^1^-CH_2_), 52.91 (Bn^2^-CH_2_), 30.61 (*p*-Cym-CH), 22.47 (*p*-Cym-CH-CH_3_), 18.25 (*p*-Cym-CH_3_); elemental analysis for C_31_H_31_BrCl_2_N_2_Ru (theoretical/found [%]): C (54.48/54.38), H (4.57/4.48) N (4.10/4.01); MS (ESI): *m*/*z* 1331.05 [M + M − 2Cl + 2H]^+^, 649.04 [M − Cl + 2H]^+^, 433.00 [M − 2Cl − *p* − Cym + H]^+^.

### 5.4. Inhibition of Bacterial TrxR (E. coli)

The TrxR (*E. coli*) inhibition assay was performed according to a previously published procedure [26]. It is partly based on the procedure developed by *Lu* et al. [32] and detects the formation of 5-TNB (5-thionitrobenzoic acid). Solutions of *E. coli* TrxR (35.4 U/mL) and of its substrate thioredoxin (Trx) *E. coli* (156 µg/mL) (both purchased from Merck (Darmstadt, Germany) and diluted with distilled water) and fresh stock solutions of the test compounds (in DMF) were prepared. TE buffer (Tris-HCl 50 mM, EDTA 1 mM, pH 7.5) containing graded concentrations of the respective compounds (20 µL) or buffer without compounds (20 µL, as control) were mixed with TrxR solution (10 µL), Trx solution (10 µL), and a solution of NADPH (200 µM) in TE buffer (100 µL) in a well on a 96-well plate. As blank solution, 200 µM NADPH in TE buffer (100 µL) mixed with a DMF/buffer mixture (40 µL) was used (final concentrations of DMF: 0.5% *v*/*v*). The solutions on the 96-well plates were incubated for 75 min at 25 °C with moderate shaking. A volume of 100 µL of a reaction mixture (TE buffer containing 200 µM NADPH and 5 mM 5,5-dithiobis-(2-nitrobenzoic acid) was added to each well to initiate the reaction. After thorough mixing, the formation of 5-TNB was monitored by a microplate reader at 405 nm in 35 s intervals (10 measurements). The values were corrected by subtraction of the blank solution’s values. The increase in concentration of 5-TNB followed a linear trend (r² ≥ 0.990) and the enzymatic activities were calculated as the slopes (increase in absorbance per second) thereof. Absence of interference with the assay components was confirmed by a negative control experiment for each test compound. The highest test compound concentration was used and the enzyme solution was replaced by TE buffer for this purpose. The IC_50_ values were calculated as the concentration of the compound decreasing the enzymatic activity of the positive control by 50%, and are presented as the means and standard deviations of three repeated experiments.

### 5.5. Antimicrobial Activity Assay

Compounds were tested in a microtiter plate assay according to CLSI guidelines against *E. coli* DSM 30083, *A. baumannii* DSM 30007, *P. aeruginosa* DSM 50071, *B. subtilis* DSM 402, *S. aureus* DSM 20231, *S. aureus* ATCC 43300 (MRSA), and *C. albicans,* as described previously [33]. *E. coli*, *A. baumannii*, *S. aureus*, and *B. subtilis* were grown in Mueller Hinton broth, *P. aeruginosa* in cation-adjusted Mueller Hinton II, and *C. albicans* DSM1386 in Sabouraud broth. Compounds were dissolved in DMSO at 10 mg/mL. Starting from a 10 mg/mL stock solution, serial dilutions in culture media were prepared with the Tecan Freedom Evo 75 liquid handling workstation (Tecan, Männedorf, Switzerland), covering a range from 512 to 0.5 µg/mL. Assay volumes were 200 µL per well. After inoculating with 5 × 10^5^ bacteria/mL from late exponential cultures grown in the same media, cultures were incubated for 16–18 h at 37 °C. The lowest compound concentration inhibiting visible bacterial growth was recorded as MIC.

## Figures and Tables

**Table 1:** MIC values (µM) against Gram-negative bacteria (*E. coli* DSM 30083, *A. baumannii*, *P. aeruginosa*), Gram-positive bacteria (*B. subtilis* 168, *S. aureus* DSM 20231, *S. aureus* ATCC 43300), and *C. albicans* DSM 1386.

	Gram-Negative			Gram-Positive			
	*E. coli*	*A. baumannii*	*P. aeruginosa*	*B. subtilis*	*S. aureus* DSM	*S. aureus* ATCC	*C. albicans*
Ciprofloxacin	1.5	24	1.2	24	12	12	-
**2a**	1349.9	1349.9	1349.9	168.7	42.2	42.2	1349.9
**2b**	1301.7	1301.7	1301.7	650.9	81.4	162.7	1301.7
**2c**	1250.8	1250.8	1250.8	78.2	19.5	19.5	1250.8
**2d**	1288.7	>1288.7	>1288.7	322.2	161.1	161.1	>1288.7
**2e**	1242.6	1242.6	1242.6	155.3	77.7	77.7	1242.6
**2f**	>1117.4	>1117.4	>1117.4	279.4	139.7	69.8	>1117.4
**3a**	>846.9	423.4	211.7	52.9	26.5	26.5	>846.9
**3b**	827.7	413.8	413.8	25.9	25.9	25.9	>827.7
**3c**	>806.8	>806.8	>806.8	25.2	25.2	25.2	>806.8
**3d**	822.4	205.6	>822.4	25.7	25.7	25.7	>822.4
**3e**	401.2	>802.4	>802.4	25.1	25.1	12.5	>802.4
**3f**	374.6	>749.1	>749.1	11.7	23.4	11.7	>749.1

## Data Availability

Not applicable.
